# Novel 3-D printed radiation shielding materials embedded with bulk and nanoparticles of bismuth

**DOI:** 10.1038/s41598-022-16317-w

**Published:** 2022-07-21

**Authors:** M. Elsafi, M. A. El-Nahal, M. I. Sayyed, I. H. Saleh, M. I. Abbas

**Affiliations:** 1 Physics Department, Faculty of Science, Alexandria University, Alexandria, 21511 Egypt; 2 Department of Environmental Studies, Institute of Graduate Studies and Research, Alexandria University, Alexandria, Egypt; 3 Department of Physics, Faculty of Science, Isra University, Amman, Jordan

**Keywords:** Materials science, Physics

## Abstract

In the present study, a new type of radiation shielding material was developed by using a 3-D printing technique which enables to create a light radiation shielding materials of a great variety of shapes and dimensions. Micro and nano bismuth particles were incorporated as a filler between the inner layers of polylactic acid thermoplastic polymer (PLA Plastic) designed of the investigated 3-D printed prototypes to achieve the desired radiation attenuation. The effect of particle size on the attenuation parameters were studied over the energy range from 0.0595 to 1.41 MeV. The mass and thickness needed to reduce the intensity of the incoming radiation to half of its original value were determined experimentally for pure polymer (ABS Plastic), polymer with bulk Bi, and polymer with nano Bi. The results reveal that bismuth NPs with average particle size of about 17 ± 3 nm have a greater mass attenuation capability than normal bulk bismuth particles, meaning they are more efficient and a lighter shield can be produced. The enhanced shielding ability of nano bismuth particles was contributed to the excellent particle distribution, leading to an increase in the probability of photons interacting with the bismuth atoms. The bismuth NPs 3-D printed objects can be considered as a promising radiation shielding candidates and also could be utilized in manufacturing of radiation medical phantom.

## Introduction

Radiation is strongly utilized in medicinal disciplines such as diagnostic radiology, radiotherapy and medical medicine. Energy generation, agriculture, manufacturing, and many others also can be considered as fields of radiation applications. Ionizing radiation, such as X-rays and gamma-rays, has enough energy to remove electrons from atoms in the human body, which can develop adverse harmful effect if not effectively attenuated. In order to mitigate these undesired effects, shielding materials are designed to eliminate a considerable portion of incoming photons through absorption process. The attenuation capability of a material varies depending on the energy and the type of radiation it may interact with. However, as a basic rule of thumb, high-Z and high-density elements are often desirable within shields because their great efficiency to attenuate high energy radiation^1–6^.

Lead has a high density, low cost, and has great attenuation near its k-absorption edge. However, lead has a major drawback due to its adverse environmental impact as well as being heavy in applications such as lead aprons. As a result, researchers have devoted their attention in finding alternatives to lead that can safely be used across various fields, and especially in medicine. Some of these substitutes include tungsten and bismuth^7,8^. Aside from bulk lead, pure polymer and polymer based composites have also been used as radiation shields, especially for non-ionizing radiation^9–12^. By introducing micro- and nanoparticles into the polymers, the radiation shielding efficiency of the polymers increases. Nanoparticles have been found to provide the greatest benefit because of the resulting greater surface-area-to-volume ratio, which leads to greater photon absorption. These fillers can also improve their mechanical, electrical, optical and chemical properties. Metal oxides such as PbO, WO_3_, Gd_2_O_3_, and Bi_2_O_3_ have been investigated as fillers in polymers used in radiation shielding applications. By introducing these oxides into the polymers^13–16^.

Much literature has been conducted to evaluate the effect grain size for varying metal and metal oxide particles. Holynska^17^ found that the impact of the particle size on the shielding ability of the samples greatly diminishes as energy increases, meaning that smaller particles perform better against lower energy photons. Additionally, when testing WO_3_, PbO, and Bi_2_O_3_ micro and nanoparticles, it was discovered that Bi_2_O_3_ nanoparticles and PbO microparticles have the best mass attenuation coefficients, which correlates with the best shielding ability^18^. Li et al.^19^, compared micro and nano gadolinium oxide Gd_2_O_3_ and found that the nano-Gd_2_O_3_ composites were more effective against X-rays and gamma-rays than the microparticles Gd_2_O_3_ composites by around 28%. Other studies have included the bulk versions of their metal oxides as another point for comparison. El-Khatib et al.^20^, studied high density polyethylene (HDPE) and tested the shielding abilities of pure HDPE, HDPE with micro CdO, and HDPE with nano CdO. They concluded that the HDPE with nano CdO has the best shielding ability. Zakaly et al.^21^ and El-Sharakawy et al.^22^ have developed new nano-composites radiation shielding materials prepared from polypropylene (PP) with CdO-NPs dispersed with different concentrations and bentonite containing Bi_2_O_3_ NPs additive, respectively. Studies have focused on enhancing the radiation-shielding properties by increasing the content of nanoparticles.

In this work, the radiation protection properties were experimentally studied using a HPGe detector and different point sources of bismuth metallic microparticles and nanoparticles used as fillers in PLA plastic matrix by using 3D printing technique. This new material created through this technique is efficient and lightweight for easy shielding as well as easy to design samples in a variety of shapes and sizes, so it can be used to manufacture medical radiation phantom by controlling bismuth concentration and designing the object.

## Materials and methods

### Samples design and manufacturing

The fabrication of 3D-printers plastics embedded with nano-bismuth can be achieved by dispersing the bismuth into plastic filament or by introducing the bismuth particles inside the unfilled designed layers of the printed objects. In order to produce filament embedded with bismuth, ABS plastic should be used since the ABS plastic and bismuth have close melting temperature, so the bismuth can be fused into ABS in the extruder machine (during manufacturing the filament), on the other hand PLA plastic is more easy to be printed than ABS and has greater density than ABS therefore PLA with bismuth as filler has been selected in the present study.

The shape of the object and the plastic shells and bismuth layers were designed by 3D-printers software “Ultimaker Cur” which is easy to use powerful software enable designers to draw, design and control the 3D-printers. This software was operated by dividing designed model file into layers and generating a printer-specific g-code which can be sent to the printer to create the desired physical object. The selected shape has been chosen to be simple cylindrical shape with dimension of 2 cm as diameter and 1 cm as height with plastic as 20% of volume of the object and 80% volume as unfilled space which will be filled by bismuth metal particles, three shells of plastics at the top, bottom and around the object that represent the necessary outer envelope of 3D-printed structure.

**Figure 1:**
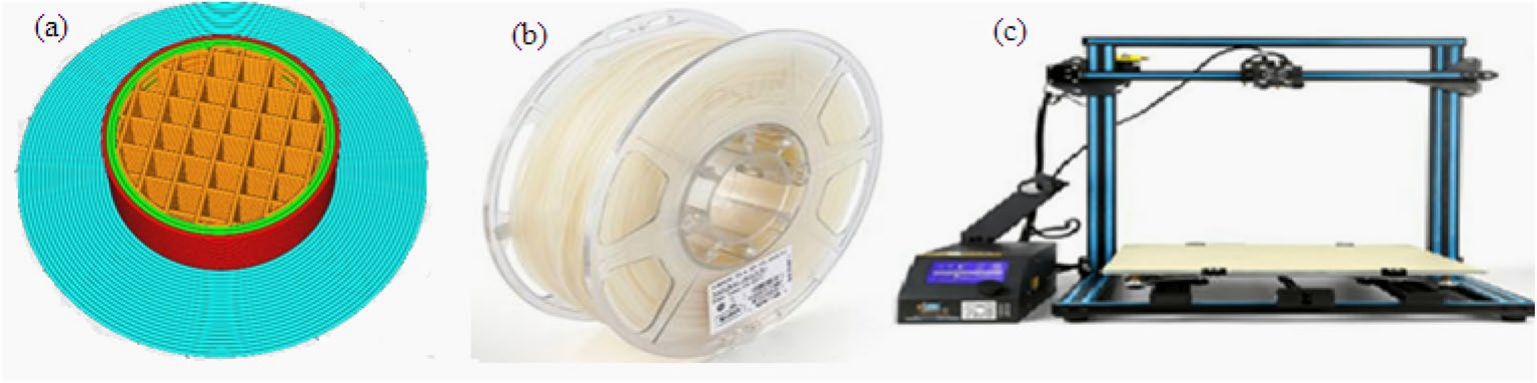
Machines used in 3D-printing, (**a**) horizontal cross section of the designed shape, (**b**) Esun Filaments and (**c**) 3D-printing machine model Creality CR10.

Figure 1a describes the designed shape and its inner layers which will be filled by bismuth. The object that produced and investigated in present study represents a prototype model, in real application any other printable shape can be manufactured and filled by bismuth particles. Used filaments were produced by the ESUN Company with diameter 1.75 mm and melting temperature 200 °C and 3D-printing machine model Creality CR10 with nozzle diameters of 0.4 mm, bed temperature of 65 °C and printing speed of 50 mm per minute (see Fig. 1b,c).

The high purity analytical grade bulk bismuth metal in the form of powder was supplied by cornel chemical laboratory in Egypt with a density of 9.8 g/cm^3^ while Nano metal bismuth particle powder were prepared by Nano tech Company in Egypt with lower density of 7.0 g/cm^3^. Bismuth metal particles have been added manually during the printing till the layers completely filled, the quantity of bulk bismuth oxide required to fill the space of the inner layers were slightly variable and more than the constant quantity of bismuth nano particles that filled same spaces of the same layers. Two sets of samples containing bismuth nano particles and bulk bismuth particles were produced; each set consists of six samples. Solid 100% filled of PLA plastic samples were also printed to represent the blank samples (without bismuth); another sample without bismuth was manufactured but with 20% to 80% ratio of plastics filled to unfilled layers ratio, in order to determine the mass of bismuth inside the samples and the densities of samples accurately.

### Samples characterization

The density of each sample was calculated theoretically due to the regular and constant geometry of printed objects as well as it was measured experimentally by Archimedes method using distilled water as the immersion fluid for confirmation by using the following relation:

1 $$\uprho = \uprho\text{L}\frac{Wa}{(Wa-Wb) },$$

where ρ_L_ is the density of the immersion liquid (density of distilled water is 1.000 g/cm^3^), W_a_ and W_b_ are the weight of samples in air and the immersion fluid, respectively. The compositions, masses and densities of the prepared samples are tabulated in Table 1. The initial thickness of the samples was 1 cm; other values can be obtained by making a combination of multiple samples of the same type.

**Table 1:** The compositions and densities of the produced samples.

Samples	Average composition wt%			Average density (g/cm^3^)
	PLA	Bulk Bi	Nano Bi	
PLA-bulk Bi	10	90	–	2.202 ± 0.005
PLA-nano Bi	32	–	68	0.704 ± 0.007
Blank (pure PLA)	100	–	–	1.303 ± 0.003

**Figure 2:**
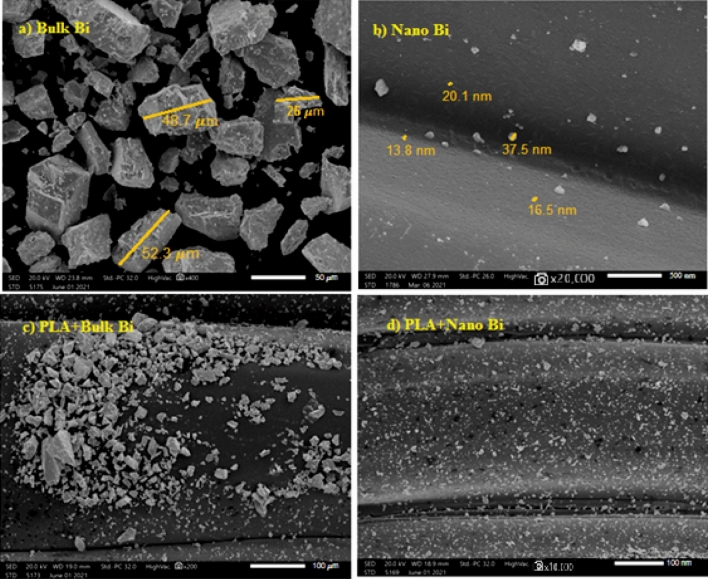
SEM images for (**a**) micro Bi, (**b**) nano Bi, (**c**) PLA with micro Bi, (**d**) PLA with nano Bi.

A scanning electron microscope (SEM) (JSM-5300, JEOL) was utilized, and the samples were cut to be exposed to the electron beam and fixed with a double coated carbon tap, which also dissipated the electron beam charge and heat buildup. The samples were covered with a fine layer of gold under a vacuum before the SEM observation, using an ion sputtering coating device (JEOL-JFC-1100E). The SEM was operated at 25 kV at a magnification order of 35,000. The samples were examined by SEM to assess the homogeneity distribution of the bismuth particle for both bulk and nano Bi inside the layers of PLA as well as to determine the average particle size of Bi inside the samples, as shown in Fig. 2.

Spherical-shaped bismuth nanoparticles with an average size of 17 ± 3 nm were achieved and demonstrated by SEM as shown in Fig. 2b, while the average size of bulk bismuth was 50 ± 8 nm. The morphological results shown in Fig. 2c,d indicated a clear difference in the distribution of nanoparticles rather than micro in PLA, where it was found that nanoparticles are more distributed and homogeneous, which improves its shielding properties.

### Gamma-rays attenuation test

**Figure 3:**
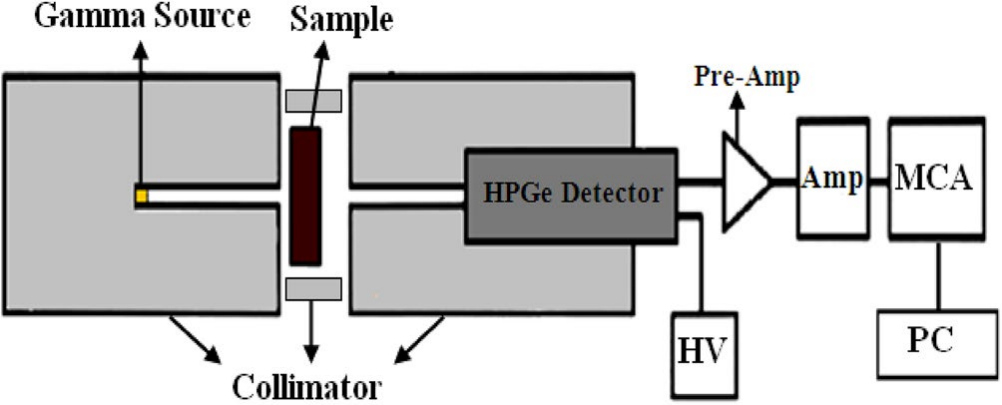
The arrangement of attenuation experiment.

The attenuation coefficients were determined experimentally by using a narrow beam technique^23^, the collimated beams of gamma photons of different energies penetrate the sample, the transmitted photons counted by using Canberra High Purity Germanium detector (HPGe) of the model: CS20-A31CL, coupled with multichannel analyzer (MCA). The detector relative efficiency was 24.5% for 1333 keV of Co-60-line. The characteristic of Eu-152 radioactive point source that utilized in this experiment in order to produce gamma photons with different energies are listed in the Table 2. The arrangement of attenuation experiment was shown in Fig. 3. The initial intensity (without sample) and transmitted intensity of each gamma line of interest were counted for a fixed time by evaluating the counts under the photo peak which represents the intensity of gamma rays. The counting time had been selected to acquire at least 10^5^ counts under each peak so that the statistical uncertainty kept less than 1%. The spectrum was processed by the Genie 2000 data acquisition and analysis software made by Canberra. A proper energy and efficiency calibrations had been done before to the measurement process.

**Table 2:** The characteristic of Eu-152 radioactive point source.

Energy (keV)	PTB nuclide	Activity (kBq)	Emission probability
121.7	Eu-152	290 ± 4.0	28.37
244.7			7.53
344.3			26.57
778.9			12.97
964.1			14.63
1086.0			13.42
1112.0			13.54
1408.1			20.85

The linear attenuation coefficient (LAC) represents the probability of photon interaction through a certain distance inside material and can be expressed by the following relation^24,25^:

2 $$ LAC = \frac{{{\text{Ln}}(I_{0}/I)}}{x}, $$

where, $I_{0} ,\,I\;{\text{and}}\;x$ represent the initial intensity, transmitted intensity and the absorber thickness, respectively. The thickness and mass needed to reduce the intensity of the incoming radiation to half of its original value called the half value layer (HVL) and the half mass value (HMV), respectively and given by the following equation^25^:

3 $$HVL= \frac{Ln 2}{LAC} ,\quad HMV= \pi {R}^{2} \times HVL\times \rho ,$$

where, $\rho \;{\text{and}}\;R$ are the density and the radius of sample material, respectively. Similarly, The thickness and mass needed to reduce the intensity of the incoming radiation to tenth of its original value called the tenth value layer (TVL) and the tenth mass value (TMV), respectively and given by the following equation:

4 $$TVL= \frac{Ln 10}{LAC} ,\quad TMV= \pi {R}^{2} \times TVL\times \rho .$$

The shielding efficiency of an absorber sample can be investigated using a parameter called the radiation protection efficiency (RPE) and given by the next equation^26,27^:

5 $$RPE \left(\%\right)=\left[1-\frac{I}{{I}_{0}}\right]\times 100.$$

## Results and discussion

Table 3 lists the linear experimentally determined attenuation coefficients (LAC) for all the tested samples between 121.7 and 1408 keV. To determine the accuracy of these measurements, the experimental LAC values for the blank polymer (the polymer without any Bi) was compared against the LAC values obtained from the XCOM database. By comparing the results from these two methods, the accuracy of the experimental method can be assessed. The percent difference between these two methods was within ± 5% deviation, proving the accuracy of the experimental data. The greatest deviation occurred at 1086 keV and is equal to a 4.85% difference. It is vital to first ensure the precision of the results for the blank polymer since XCOM cannot predict the LAC for the sample that contains nanoparticle Bi. Once the results for the blank polymer are determined to be accurate within a desirable deviation, the same experimental setup can be used to assess the samples with bulk and nanoparticle Bi without worrying about the reliability of the process. Furthermore, the table lists the LAC values of bulk and nano Bi to compare them against each other. These values demonstrate that the LAC of bulk Bi is greater than the blank and nano Bi samples. For examples, at 344.3 keV, the blank polymer has an LAC equal to 0.129 cm^−1^, the bulk Bi sample has an LAC equal to 0.677 cm^−1^, and the nano Bi sample has an LAC equal to 0.493 cm^−1^. The bulk Bi sample has the greatest LAC because it has the greatest density out of the three investigated samples.

**Table 3:** The LAC for different investigated samples and the pure PLA polymer (blank) compared with the XOM results and its uncertainty.

Energy (keV)	LAC (cm^−1^)		∆ (%)	LAC (cm^−1^)	
	Blank (XCOM)	Blank (exp)		With bulk Bi	With nano Bi
121.7	0.1742	0.1762 ± 0.0013	1.09	1.9811 ± 0.0009	1.6692 ± 0.0013
244.7	0.1415	0.1394 ± 0.0015	− 1.41	1.0472 ± 0.0015	0.7333 ± 0.0017
344.3	0.1252	0.1291 ± 0.0010	3.53	0.6771 ± 0.0019	0.4930 ± 0.0014
778.9	0.0883	0.0864 ± 0.0018	− 3.20	0.1814 ± 0.0010	0.1344 ± 0.0009
964.1	0.0804	0.0835 ± 0.0020	4.41	0.1223 ± 0.0013	0.0991 ± 0.0021
1086	0.0755	0.0795 ± 0.0013	4.85	0.1050 ± 0.0008	0.0843 ± 0.0018
1112	0.0751	0.0782 ± 0.0009	3.98	0.0991 ± 0.0011	0.0811 ± 0.0011
1408	0.0662	0.0641 ± 0.0012	− 2.43	0.0852 ± 0.0014	0.0713 ± 0.0010

**Figure 4:**
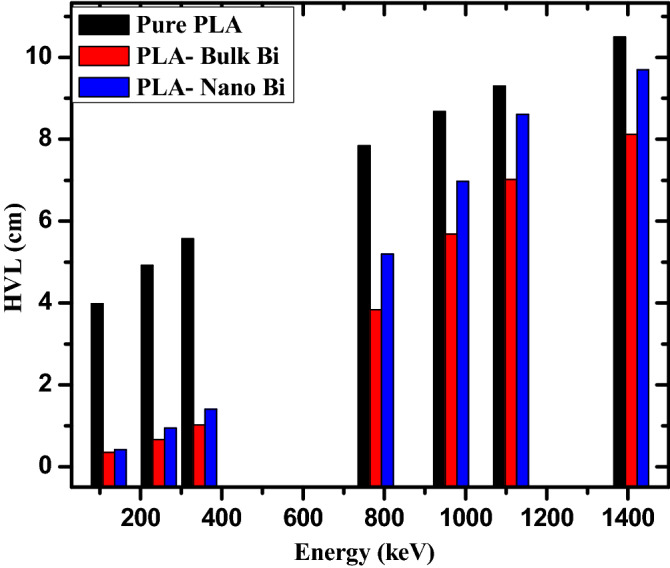
The HVL for the pure PLA, PLA with bulk Bi, and PLA with nano Bi at different energies.

In Fig. 4, the half value layer (HVL) for the pure polymer, polymer with bulk Bi, and polymer with nano Bi were determined in the same energy range used for the LAC values. Both the polymers containing Bi have a lower HVL than the pure polymer, which means that the addition of Bi leads to a decrease in the thickness of the sample needed to attenuate the intensity of the incoming photons to half of their original value. Additionally, it can be seen that the effect of Bi on the HVL values is clearer at lower energies than at higher energies. For example, at 121.7 keV, the HVL values are equal to 3.981, 0.350, and 0.415 cm for the pure polymer, polymer with bulk Bi, and polymer with nano Bi respectively. Meanwhile, at 1408 keV, the HVL values are equal to 10.491 cm, 8.123 cm, and 8.699 cm, respectively. This result signifies that the radiation shielding advantage of the polymers with a Bi decrease as the energy increases. When comparing the HVL values of the bulk and nanoparticle polymers, it can be observed that the HVL for the bulk Bi polymer is lower than the nanoparticle Bi_,_ which is related to the greater density of the bulk Bi sample, lowering HVL.

**Figure 5:**
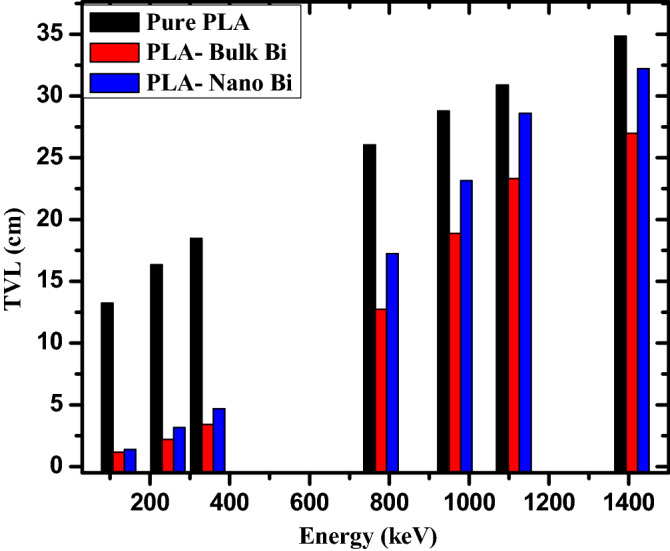
The TVL for the pure PLA, PLA with bulk Bi, and PLA with nano Bi at different energies.

The tenth value layer (TVL) of a sample is defined as the thickness of the material needed to reduce the intensity of the incoming radiation to one-tenth of its original value. Figure 5 illustrates the TVL of the three tested polymers. Since TVL is closely related to HVL, the same trends can be observed in both figures. As the incoming photon energy increases, the TVL values all increase with it. The TVL of the pure polymer sample, for example, increases from 12.226 cm at 121.7 keV to 18.479 cm at 344.3 keV, 28.810 cm at 964.1 cm, and 34.851 cm at 1408 keV. In addition, the TVL values following the order of: TVL_pure polymer_ > TVL_nano Bi2O3_ > TVL_bulk Bi2O3_. At 778.8 keV, for instance, the pure polymer has a TVL equal to 26.046 cm, the nano Bi has a TVL equal to 17.238 cm, and the bulk Bi has a TVL equal to 12.743 cm. The order of these values is still correlated with the densities of the samples. Since the polymer with bulk Bi has the lowest TVL at all tested energies, this sample is the most space-efficient and has the greatest potential for radiation shielding applications. The discrepancy between the TVL results also decreases with increasing energy. At the lowest tested energy, the lowest and greatest TVL has a difference of 12.063 cm, while at the highest energy their difference is equal to 7.866 cm, it should be noted that since TVL requires greater attention than HVL, the TVL values are all greater than their respective HVL values.

**Figure 6:**
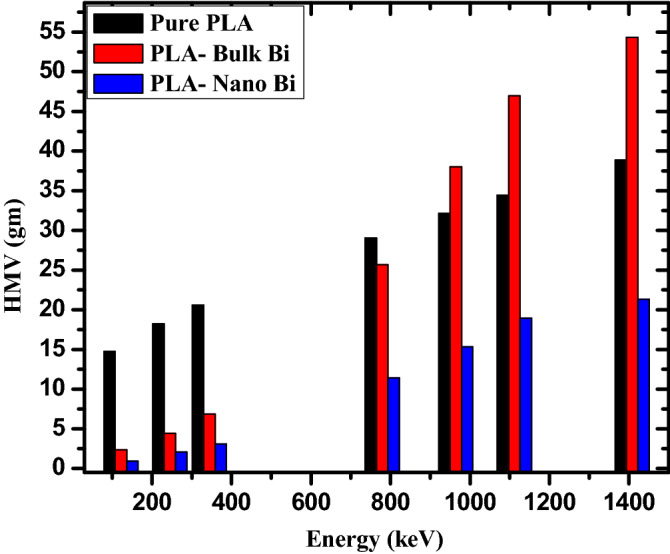
The HMV for the pure PLA, PLA with bulk Bi, and PLA with nano Bi at different energies.

The mass of a material needed to reduce the intensity of the incoming radiation to half of its original value is defined as its HMV. The HMV of the three investigated samples are graphed in Fig. 6 at the first four energies, the HMV of the pure polymer is greater than the HMV for the bulk and nano Bi samples. For example, at 244.7 keV, the pure polymer has an HMV equal to 18.22 g, while the polymer with bulk Bi has an HMV equal to 4.43 g and the nano Bi polymer has a HMV equal to 2.08 g. As energy further increases, however, (E ≥ 1086 keV) the bulk Bi polymer has the greatest HMV. At 1086 keV, for instance, the HMV values are equal to 113.11 g, 146.14 g, and 60.34 g for the pure polymer, polymer with bulk Bi_,_ and polymer with nano Bi, respectively. These results indicate that at all energies the polymer with nano Bi has a much lower HMV than the other samples, which is greatly important for real applications. In other words, a much lighter sample can be used to attenuate the same amount of photons, making this shield more practical and viable as a portable or movable radiation shield. In contrast, the polymer with bulk Bi offers great attenuation, but is much heavier than the nano Bi_,_ making it impractical for some applications.

**Figure 7:**
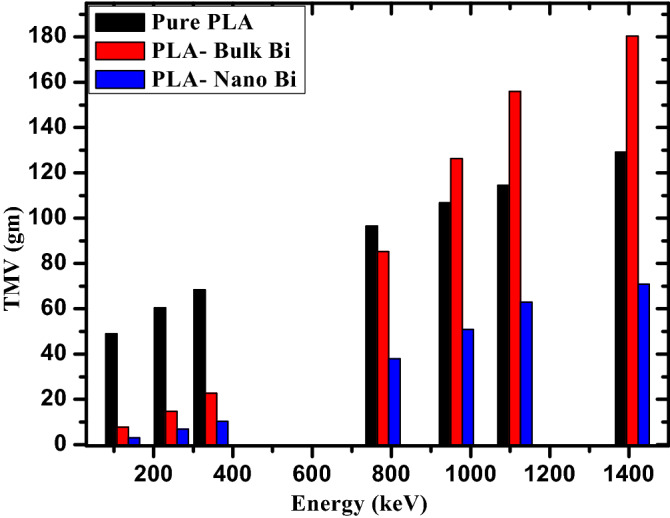
The TMV for the pure PLA, PLA with bulk Bi, and PLA with nano Bi at different energies.

Like HMV, TMV is the counterpart where the intensity of radiation is reduced to a tenth of its original value. This naturally results in the TMV values being much greater than the HMV results. Figure 7 illustrates the TMV of the three investigated polymers. Due to the similarity between the two parameters, the same trends can be observed in both figures. First, the TMV of the polymers increase with increasing energy. For example, the TMV of the polymer with bulk Bi increases from 7.78 g at 121.7 keV to 22.76 g at 334.3 keV, 126.27 g at 964.1 keV, 155.97 g at 1112 keV, and 180.48 g at 1408 keV. At energies below 1086 keV, the TMV follow the order of pure polymer > bulk Bi > nano Bi. For instance, at 344.3 keV, the pure polymer has a TMV of 68.47 g, the polymer with bulk Bi has a TMV of 22.76 g, and the polymer with nano Bi has a TMV of 10.27 gm. At energies greater than 1086 keV, however, the TMV of the bulk Bi sample surpasses the pure polymer. At 1112 keV, the pure polymer has a TMV equal to 114.45 g and the bulk Bi sample has a TMV of 155.97 g. This figure reaffirms the conclusion that the nano Bi is more practical for real world applications due to the much lower mass needed to attenuate the same amount of photons, even if a greater thickness might be needed compared to bulk Bi. Therefore, when selecting the most desirable radiation shield for a specific application, both the thickness and the mass of the shield must be carefully considered and compared against each other.

**Figure 8:**
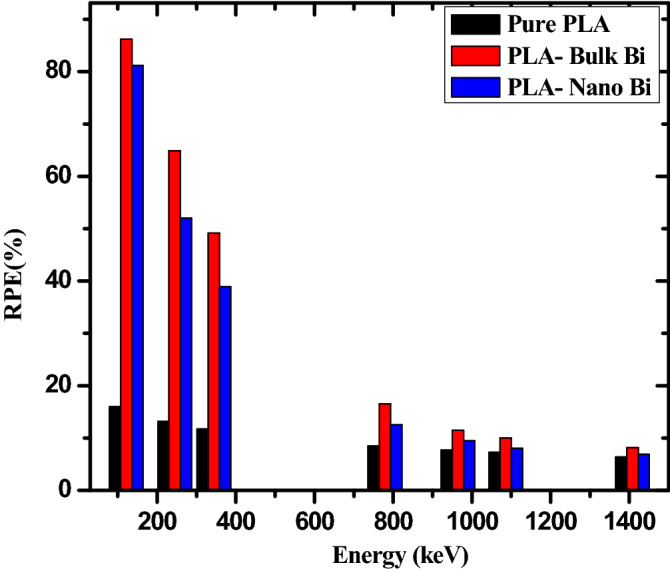
The RPE for the pure PLA, PLA with bulk Bi, and PLA with nano Bi at different energies.

Figure 8 illustrates the radiation protection efficiency (RPE) of the three investigated samples as a function of the incoming photon energy. This figure demonstrates that RPE decreases as energy increases for all three samples. The maximum RPE occurs at the lowest tested energy, 121.7 keV, and is equal to 86.20% for the polymer with bulk Bi2O3. RPE, then quickly decreases to its minimum value at the highest tested energy. Continuing with this sample, its RPE is equal to 64.91% at 244.7 keV, 16.53% at 778.9 keV, 10.00% at 1086 keV, and 8.18% at 1408 keV. This sharp drop signifies that the samples have a good attenuation capability at lower photon energies, and that their abilities to absorb radiation decreases with increasing energy. Thus, to maintain the same amount of attenuation against higher energy photons, the thickness of the polymer must be increased if the application requires it. Furthermore, the figure shows that both the polymers with Bi2O3 have a higher RPE than the pure polymer. For example, at 964.1 keV, the pure polymer has an RPE of 7.68%, while the polymer with bulk Bi2O3 has an RPE of 11.48%, and the polymer with nanoparticle Bi2O3 has an RPE of 9.47%. This result suggests that the addition of Bi2O3 enhances the RPE of the tested samples.

Finally, the present work was compared with an essential material used for gamma ray shielding such as glass and concrete as well as previous works related to our work such as HDPE embedded in CdO nanoparticles and silicon rubber (SR) embedded with Bi_2_O_3_ nanoparticles. The comparison was reported in Table 4 and it was clear that the results of the current work provided light shielding materials with a good attenuation parameters compared to the other scheduled results.

**Table 4:** Comparison of the present work with other previously reported shielding materials.

Shielding Materials	Density (g cm^3^)	MAC, cm^2^ g^−1^			HVL, cm			References
		122 keV	344 keV	1408 keV	122 keV	344 keV	1408 keV	
Bismuth-borate glass composites with 80%B_2_O_3_ and 20%Bi_2_O_3_	2.89	0.727	0.138	0.052	0.330	1.738	4.612	Al-Hadeethi et al.^28^
Ordinary concrete	2.34	0.115	0.075	0.056	2.576	3.950	5.290	El-Nahal et al.^29^
Concrete + 15% Bi_2_O_3_ NPs	2.78	0.395	0.125	0.060	0.631	1.995	4.156	
HDPE	0.94	0.161	0.115	0.061	4.580	6.412	12.088	El-Khatib et al.^30^
HDPE + 10%CdO NPs	1.11	0.253	0.130	0.064	2.468	4.804	9.757	
HDPE + 40%CdO NPs	1.57	0.468	0.134	0.062	0.943	3.295	7.121	
Silicon rubber (SR)	1.19	0.206 (81 keV)	0.113 (356 keV)	0.062 (1333 keV)	2.828 (81 keV)	5.155 (356 keV)	9.395 (1333 keV)	Abbas et al.^31^
SR + 5% Bi_2_O_3_ NPs	1.30	0.327 (81 keV)	0.125 (356 keV)	0.063 (1333 keV)	1.631 (81 keV)	4.266 (356 keV	8.463 (1333 keV)	
SR + 30% Bi_2_O_3_ NPs	1.71	1.025 (81 keV)	0.189 (356 keV)	0.064 (1333 keV)	0.395 (81 keV)	2.145 (356 keV)	6.334 (1333 keV)	
Blank PLA	1.30	0.135	0.100	0.045	3.950	5.332	11.849	Present work
PLA-bulk bismuth	2.20	0.900	0.307	0.039	0.350	1.026	8.079	
PLA-nano bismuth	0.70	2.384	0.704	0.101	0.415	1.407	9.804	

## Conclusion

The designed prototypes were designed and created using 3D printing method and this method proved to be capable of creating excellent radiation shielding candidates and good replacements to already available shields based on lead in real applications. Moreover, the suggested technique allows manufacturers to create radiation shields with a large variety of shapes and dimensions that can fit any the requirements of any application. Additionally, the low density nano bismuth particles have a better mass attenuation coefficient than bulk bismuth, which can help develop a lighter and more effective radiation shielding material. This advantage is attributed to the homogenous distribution of nanoparticles with approximately equal size within the printed object layers. This novel technique can be applied to a wide variety of radiation shielding applications from the shielding of low energy X-ray diagnostic to the environmental shielding of nuclear reactor due to its proved great ability of radiation attenuation and being consisting of both light and heavy material and also can be used to produce medical phantoms.

## Data Availability

All data generated or analyzed during this study are included in this published article.
